# Gait variability of outdoor vs treadmill walking with bilateral robotic ankle exoskeletons under proportional myoelectric control

**DOI:** 10.1371/journal.pone.0294241

**Published:** 2023-11-13

**Authors:** Rachel Hybart, Daniel Ferris

**Affiliations:** J. Crayton Pruitt Department of Biomedical Engineering, University of Florida, Gainesville, Florida, United States of America; University of Verona: Universita degli Studi di Verona, ITALY

## Abstract

Lower limb robotic exoskeletons are often studied in the context of steady-state treadmill walking in laboratory environments. However, the end goal of these devices is often adoption into our everyday lives. To move outside of the laboratory, there is a need to study exoskeletons in real world, complex environments. One way to study the human-machine interaction is to look at how the exoskeleton affects the user’s gait. In this study we assessed changes in gait spatiotemporal variability when using a robotic ankle exoskeleton under proportional myoelectric control both inside on a treadmill and outside overground. We hypothesized that walking with the exoskeletons would not lead to significant changes in variability inside on a treadmill or outside compared to not using the exoskeletons. In addition, we hypothesized that walking outside would lead to higher variability both with and without the exoskeletons compared to treadmill walking. In support of our hypothesis, we found significantly higher coefficients of variation of stride length, stance time, and swing time when walking outside both with and without the exoskeleton. We found a significantly higher variability when using the exoskeletons inside on the treadmill, but we did not see significantly higher variability when walking outside overground. The value of this study to the literature is that it emphasizes the importance of studying exoskeletons in the environment in which they are meant to be used. By looking at only indoor gait spatiotemporal measures, we may have assumed that the exoskeletons led to higher variability which may be unsafe for certain target populations. In the context of the literature, we show that variability due to robotic ankle exoskeletons under proportional myoelectric control does not elicit different changes in stride time variability than previously found in other daily living tasks (uneven terrain, load carriage, or cognitive tasks).

## Introduction

Lower limb robotic ankle exoskeletons can aid rehabilitation and augment healthy human performance, but are not yet widely adopted into daily use in the general population [1–4]. To improve performance, and subsequently increase adoption, of lower limb robotic exoskeletons it would be beneficial to include testing and validation of devices in contexts that are closer to real-world use. Validation of lower limb robotic exoskeletons typically focuses on outcomes during performance of steady-state or rhythmic tasks such as treadmill walking, repetitive loading, or stair climbing in controlled laboratory settings [2,5,6]. However, the end goal for many assistive and augmentative lower limb robotic exoskeletons is integration into everyday life, which includes navigating complex tasks and environments. Recent research has found that increasing variability of walking can lead to faster optimization by the nervous system to control gait mechanics when walking with an energy damping knee brace [7]. There is an increase in research on how environmental changes, such as terrain and obstacles, alter the effectiveness of exoskeletons in the real-world [8–11]. The ability of an exoskeleton to adapt with the user to different environments is important in building user trust in the exoskeleton, which is often associated with perceptions of stability and balance [12,13]. Unanticipated responses from the exoskeleton to the environment may lead to a distrust between the user and the exoskeleton and reduce the likelihood of continued use.

Gait variability is an important measure that is often ignored in the training and use of robotic lower limb exoskeletons during human locomotion. There is extensive literature on gait variability for healthy individuals and individuals with gait deficits. Researchers use gait measures, such as stride length, stride time, stance time, cadence, and swing time and the variability of those measures as indicators of healthy, typical gait [14–17]. Healthy young adults typically have smaller variations in gait spatiotemporal measures when compared to individuals with neuromuscular impairments [18], but the ability to allow higher variability to maintain stability in new, or complex environments is also indicative of healthy gait [19–21]. Introducing novel tasks, like walking with an exoskeleton, may lead to a higher cognitive load for the individual which in turn may initially raise the amount of variability in gait measurements [22–25]. Few studies have assessed how exoskeletons affect gait spatiotemporal measures, and even fewer have done so in the real-world [19,26,27]. Given the importance of variability on optimization by the nervous system when using wearable robotic devices [7,28–30], studying gait spatiotemporal measures during use of robotic lower limb exoskeletons is critical to continued development of the devices.

The purpose of this study was to determine the effect of proportional myoelectric controlled robotic ankle exoskeletons on gait variability while walking on a treadmill indoors and overground outside. Previous research on human walking indicates that gait variability is lower while walking on a treadmill compared to walking overground [7,14,31–33]. We were curious to know if we would find similar results walking overground outside and on a treadmill with a powered ankle exoskeleton. We used a custom proportional myoelectric controller on commercially available robotic ankle exoskeletons (Dephy Exoboots, Maynard, MA) similar to what has been used in past treadmill studies [34–37]. The exoskeletons aided plantarflexion by providing torque during the push off phase of stance based on the user’s soleus electromyography [38]. We chose the coefficient of variation of stride length, stride time, stance time, and swing time as our outcome measures of gait variability. We hypothesized that there would be higher variability in gait spatiotemporal measures when walking outside overground both with and without the exoskeletons compared to walking inside on a treadmill. Our second hypothesis was that walking with the exoskeletons would not lead to higher variability than walking without the exoskeletons because the controller takes into account user intention via myoelectric signals. We also tested whether there were differences in gait variability over four days of exoskeleton use. We expected that the initial training day would have the highest variability and the final testing day would have the lowest variability due to locomotor adaptation by the user.

## Methods

### Participants

We recruited 12 participants (5 female, 7 male) with the following characteristics, mean (s.d.): height: 1.73 (0.075) m; mass: 69.3 (10.2) kg; age 22.6 (8.66) yrs. The standard deviation for the age of our participants is high because of a single 50-year-old participant. None of that participant’s data, besides their age, were outliers from the group data and therefore was kept for all analyses. Participants were recruited between June and December 2022 for this study using flyers around the University of Florida campus as approved by the Institutional Review Board. For treadmill walking participants walked at an average speed of 1.14 (0.12) m/s. For the outdoor conditions participants walked at an average speed of 1.2 (0.05) m/s while walking with the exoskeletons and 1.17 (0.07) m/s when walking without the exoskeletons. Participants reported no previous neurological or musculoskeletal conditions. Each participant was right-handed and had no previous experience walking with a robotic ankle exoskeleton prior to the first day of training for this study. Each participant read and signed a written informed consent form approved by the University of Florida Institutional Review Board (IRB#201801218). Authors were aware of the identity of participants during data collection, but the data themselves were deidentified during analysis. We expected sufficient statistical power from 12 participants based on our previous research [10,35,36,39]. Preliminary statistical power analysis on pilot data for three participants indicated that 12 participants would provide 0.8 power for an alpha equal to 0.05 to test our main hypothesis on gait variability with and without the exoskeleton.

### Equipment

**Fig 1:**
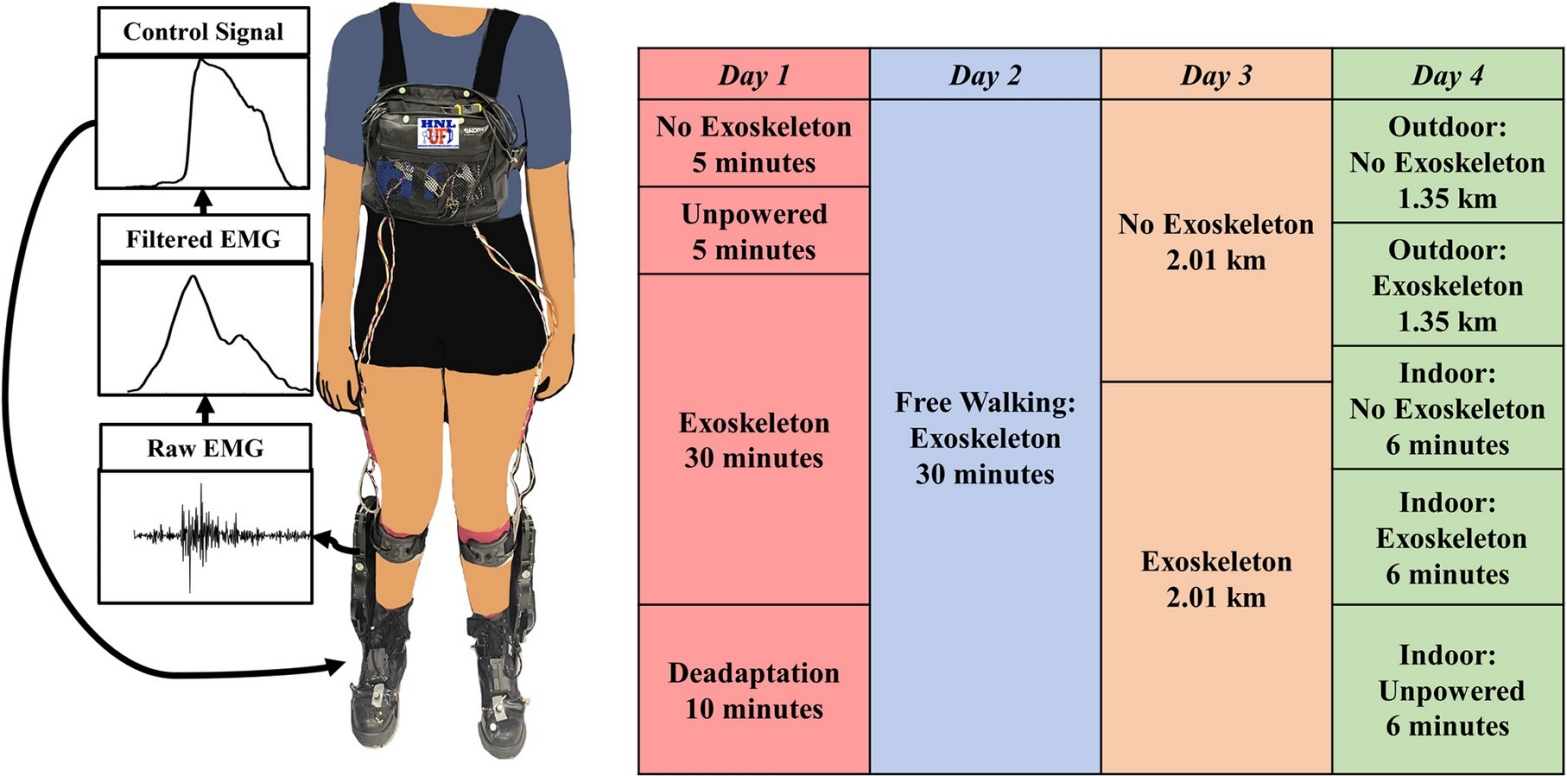
(Left) Collection setup including the Dephy Exoboots, Coapt snap electrode EMG system, and a Raspberry Pi worn by a participant. The raw EMG data were collected, filtered, and multiplied by a participant specific gain to get a control signal communicated to the Dephy ExoBoots via a USB cable. (Right) The 4-day protocol, with a total of ~106 minutes of walking with the ExoBoots powered.

We implemented a proportional myoelectric controller on the commercially available Dephy exoskeletons with an open-source software option (Dephy, Inc. Maynard, MA). A proportional myoelectric controller with an input of the user’s soleus muscle provided the input current to the motor [38]. The soleus electromyography (EMG) data were high-pass filtered (2^nd^ order Butterworth, cutoff frequency 50 Hz), full wave rectified, and low-pass filtered (2^nd^ order Butterworth, cutoff frequency 8 Hz). We then multiplied the resulting signal by a participant specific gain determined while walking with the exoskeletons unpowered to get the controlling motor current (Fig 1). Participant-specific static gains were determined during an Unpowered walking condition, and ensured the controlling current reached the maximum value of 7.6 A q-axis (phase) current, which is equivalent to 20 A of line-to-line current as described by Dephy, Inc. Participants walked for five minutes to determine their participant-specific gain. The values were adjusted until the controlling current plateaued at the maximum value on every step. This value was kept the same across testing days, as we did not see a reduction in the amount of current being supplied across testing days. The controlling current was transformed via a variable transmission ratio into an ankle torque that pulled the toe down. The exoskeleton torque is implemented by an electromechanical actuator that transmits the torque via a chain drive [40]. The exoskeletons only assisted in plantarflexion and the control signal dropped to zero during swing when the foot switches did not register any weight on the foot. The only resistance to dorsiflexion during swing was the passive unwinding of the chain and the weight of the boots. The total system, including the exoskeletons and a backpack with a raspberry pi and battery pack added 4.5 kg to the participants. We measured EMG of the soleus, tibialis anterior, and gastrocnemii on each leg using bipolar skin electrodes (Coapt, Inc. Chicago, IL). We only used the soleus EMG in the control of the exoskeletons. We used inertial measurement units (IMU) on the shanks and feet to find ankle angle, stride time, stride length, swing time and stance time (APDM, Inc. Portland, OR) [41]. IMUs were placed on the lateral longitudinal arch of the foot and the lateral shank just below the cuff of the exoskeleton. The exoskeleton shoes contained carbon fiber plates which allowed for minimal movement of the IMUs with relation to the foot in the configuration used. We used a force instrumented treadmill to validate gait events from the IMUs on the treadmill (Bertec, Co. Columbus, OH).

### Collection

Participants completed 4 days of testing with at least 48 hours between data collection days to allow for motor learning consolidation [42] (Fig 1). We chose four days based on previous research that showed participants fully adapted to proportional myoelectric control of a pneumatic exoskeleton after three days [35,36]. The fourth day was added because we wanted participants to have three days of structured use of the exoskeletons (Days 1,3, and 4) and one day of more free use of the exoskeletons (Day 2). The initial training day (Day 1), included 50 total minutes of treadmill walking at a self-selected speed, including 30 minutes with the exoskeleton powered. The self-selected speed was a set speed on the treadmill found by asking participants to adjust the treadmill speed until they found the speed most comfortable for walking. This was found before any equipment was placed on the participants. The second day of training included a 30-minute period where the participants were able to freely walk outside around the University of Florida campus to explore the limits of the exoskeleton assistance. On the third day, participants walked a 2.01 km course with and without the exoskeletons. Participants walked for approximately 30 minutes on this day. We split the course into three sections so that results would not be influenced by fatigue due to duration or the warm outdoor temperatures in Florida in the late summer. The final day (Day 4) included metabolic cost measurements taken while walking with and without the exoskeletons, both inside on a treadmill and outside. Conditions on days 3 and 4 were randomized.

### Data analysis

We found heel strikes and toe offs from IMUs on the lateral longitudinal arch of the feet. We found the peaks in vertical acceleration surrounding the near zero velocity region that indicates stance phase similar to what is described in previous research [43]. We ensured the validity of the gait events by comparing the IMU detected gait events and treadmill detected gait events during our indoor walking trials. We used those gait events to calculate stride time, stance time, and swing time. Stride lengths were calculated by converting the IMU accelerations to the global coordinate system, double integrating to find the position of the IMUs in space and calculating the difference in position from heel strike to heel strike. Stride times outside were calculated using speeds time-synced to the IMU data from a wrist GPS watch (TIMEX IRONMAN R300 GPS Smartwatch). We analyzed data for 100 strides from each condition, based on previous research showing that 100 strides is sufficient and necessary for understanding gait variability when walking overground [15]. For the 100 strides, we took the mean and standard deviation for each condition and gait parameter for each participant and calculated the averages for stride time, stride length, stance time, and swing time. For each participant we calculated the coefficient of variation (CoV) of stride time, stride length, stance time, and swing time by dividing the standard deviation by the mean and multiplying by 100 to present the data as a percentage. Previous publications focus on the metabolic and neuromechanical adaptations to this controller and exoskeleton combination [44,45], so we do not go into detail on the findings. We calculated EMG root mean square (RMS) values during stance for four muscles of each leg (tibialis anterior, medial gastrocnemius, lateral gastrocnemius, and soleus) for each condition and day. The values were then normalized to the no exoskeleton condition of that day.

### Statistical analysis

We used 2-way MANOVAs to assess differences in means and coefficient of variations in gait parameters between factors. The first used factors of Day (1 and 4) and Condition (Exoskeleton, Unpowered, and No Exoskeleton), the second used factors of Location (Treadmill and Overground) and Condition (Exoskeleton and No Exoskeleton). We did post-hoc Tukey-HSD when statistical differences were found across the Exoskeleton, Unpowered, and No Exoskeleton conditions. We did the calculations for the Day and Condition factors first, once we determined there was not a significant difference due to day, we used the data from Day 4 for statistical analyses between indoor and outdoor. We assigned a significance value of α = 0.05 for both tests. For EMG RMS values we either did 1-way ANOVAs with condition being the factor for each muscle on each day, or for the outdoor condition we performed a t-test because there were only 2 conditions. We used SPSS for statistical analysis.

## Results

**Fig 2:**
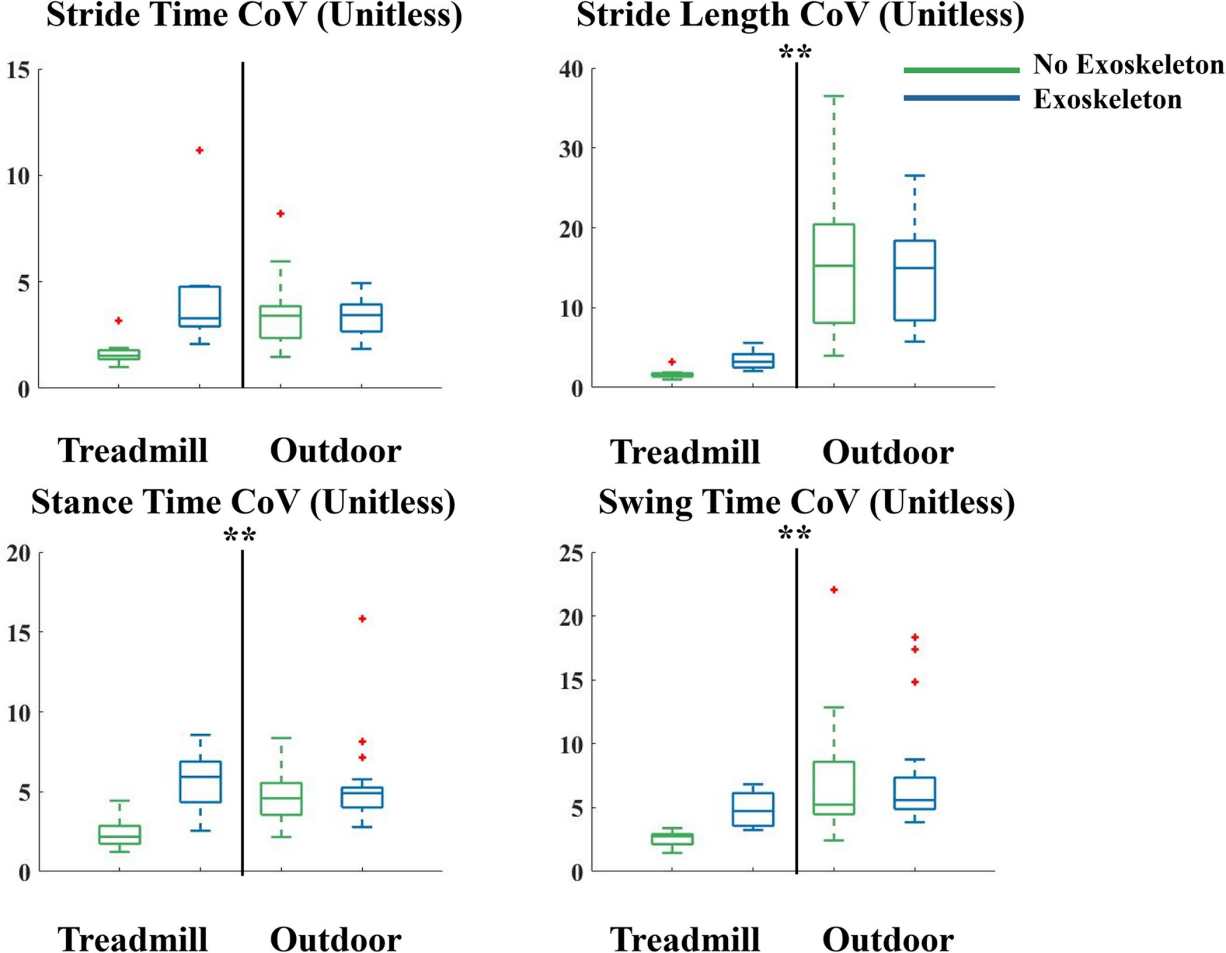
Coefficient of Variation (CoV) presented as a percentage for the stride time, stride length, stance time and stance length while walking on a treadmill (left side of each subplot) and outside overground (right side of each subplot) without (green) and with (blue) the exoskeletons. Outdoor walking had higher coefficients of variation in stride length, stance time, and swing time when compared to treadmill walking. Data is represented as a box plot where the central line indicates the median of the data, the top and bottom edges of the box indicate the 25^th^ and 75^th^ percentile, and the whiskers represent the minimum and maximum values. Red crosses indicate outliers in the data. ** indicates significant differences between treadmill and outdoor walking. There were not significant differences between the exoskeleton and no exoskeleton condition when walking outside.

Consistent with our hypothesis there were significant differences in mean and variability of gait spatiotemporal measures when walking outside over ground compared to inside on a treadmill. The coefficient of variation while walking outside compared to walking on a treadmill was significantly higher for stance time (p = 0.044), swing time (p = 0.002), and stride length (p<0.001) (Fig 2). In partial support of our hypothesis, we found no significant differences in means or coefficients of variation between exoskeleton and no exoskeleton conditions when walking outside. The coefficient of variation of the stride length was 14% in the outdoor exoskeleton condition and 3% in the indoor exoskeleton condition, a 119% difference. The coefficient of variation of stride length was 17% in the outdoor no exoskeleton condition and 2% in the indoor no exoskeleton condition, a 162% difference. The coefficient of variation of the stance time was 5% in the outdoor exoskeleton condition and 6% in the indoor exoskeleton condition, an 8% difference. The coefficient of variation of the stance time was 5% in the outdoor no exoskeleton condition and 2% in the indoor no exoskeleton condition, a 64% difference. The coefficient of variation of the swing time was 7% in the outdoor exoskeleton condition and 5% in the indoor exoskeleton condition, a 36% difference. The coefficient of variation of the swing time was 7% in the outdoor no exoskeleton condition and 3% in the indoor no exoskeleton condition, a 90% difference.

**Fig 3:**
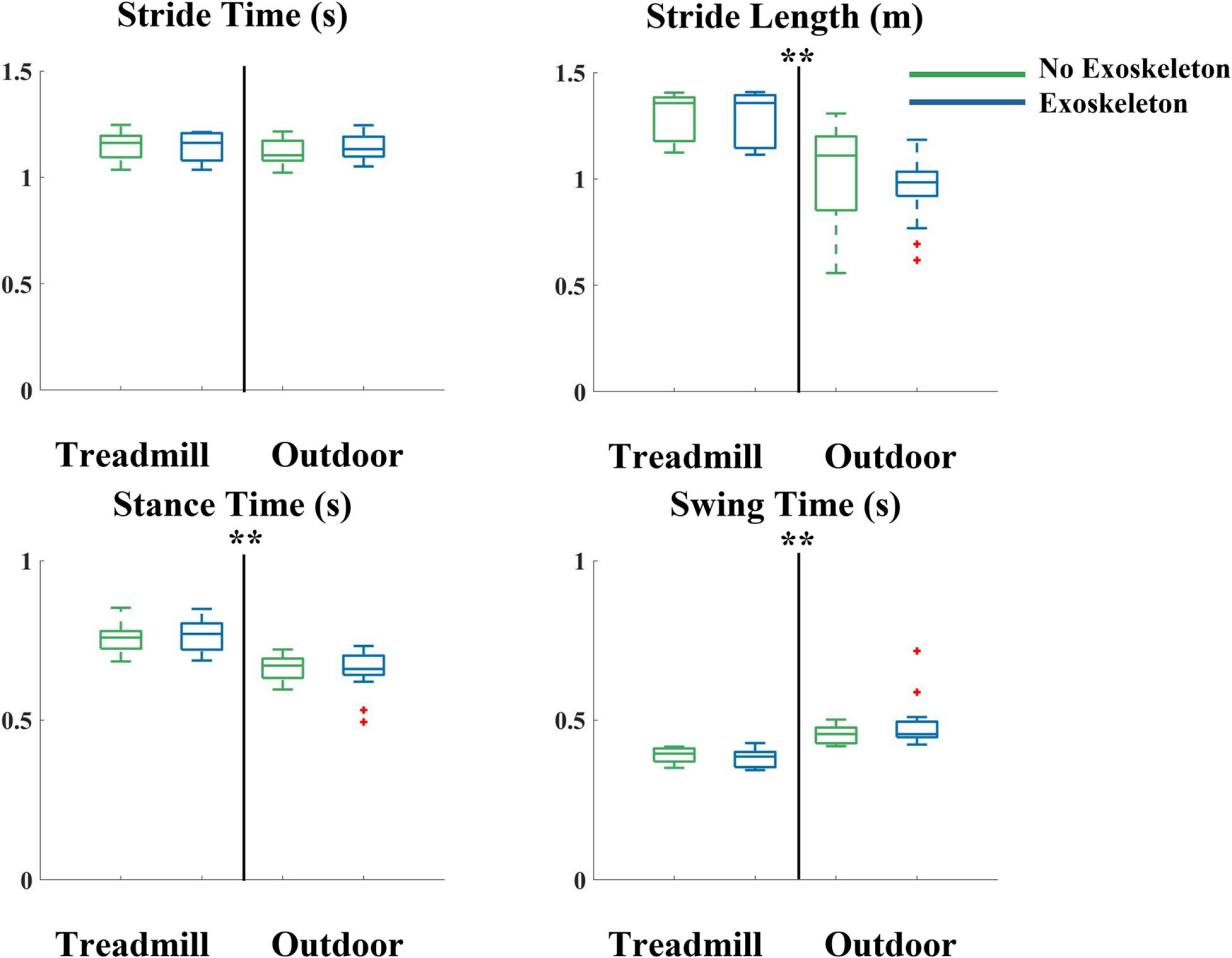
Stride time, stride length, stance time, and swing time when walking on a treadmill (left side of each subplot) and walking outside overground (right side of each subplot) without (green) and with (blue) the exoskeletons. There were significantly longer stride lengths and swing times when walking outside compared to on the treadmill. There were significantly shorter stance times when walking outside compared to on the treadmill. Data is represented as a box plot where the central line indicates the median of the data, the top and bottom edges of the box indicate the 25^th^ and 75^th^ percentile, and the whiskers represent the minimum and maximum values. Red crosses indicate outliers in the data. ** indicates significant differences due to location.

The stride length and stance time were significantly shorter outside compared to inside on a treadmill (p<0.001), and the swing time was significantly longer outside compared to inside on a treadmill (p<0.001) (Fig 3). There was no significant difference in the mean stride time outside compared to inside on a treadmill. Stride lengths were shorter outside (0.96 m with the exoskeletons and 1.01 m without the exoskeletons) compared to on the treadmill (1.29 m with and without the exoskeletons). Participants walked with shorter stance times outside (0.66 s with and without the exoskeletons) than on the treadmill (0.76 s with and without the exoskeletons). Participants walked with longer swing times outside (0.48 s with the exoskeletons and 0.45 without the exoskeletons) outside than on the treadmill (0.38 s with the exoskeletons and 0.39 s without the exoskeletons).

**Fig 4:**
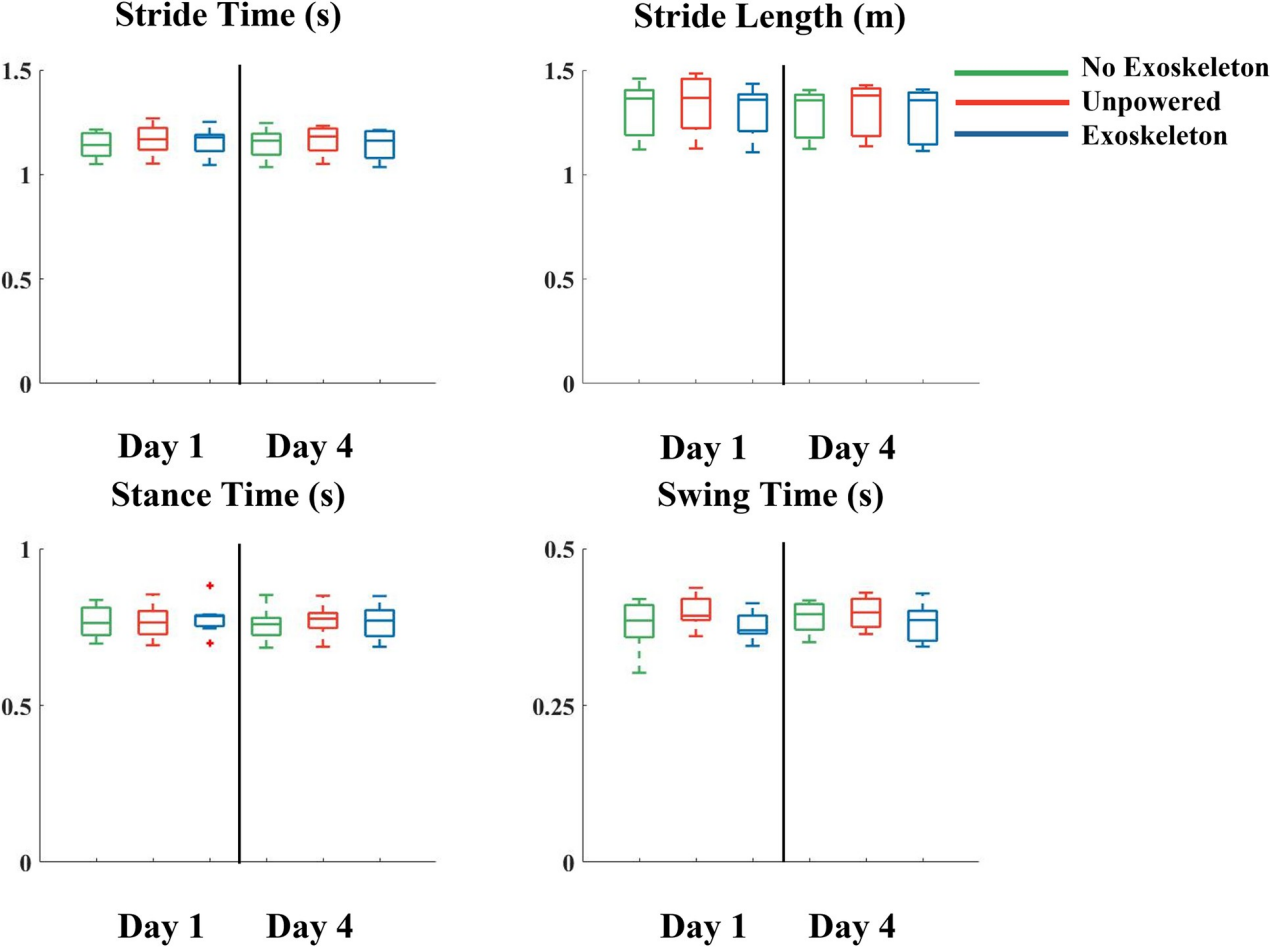
Mean stride time, stride length, stance time, and swing time when walking on a treadmill on the first (left side of each subplot) and final (right side of each subplot) days of training without the exoskeletons (green), with the exoskeletons on and unpowered (orange), and with (blue) the exoskeletons. There were no changes in stride time, stride length, stance time, or swing time from the first day of training to the final day of training. Data is represented as a box plot where the central line indicates the median of the data, the top and bottom edges of the box indicate the 25^th^ and 75^th^ percentile, and the whiskers represent the minimum and maximum values. Red crosses indicate outliers in the data.

There were no significant differences in stride time, stance time, stride length, or swing time between the no exoskeleton, unpowered, and exoskeleton conditions or between day 1 and day 4 (Fig 4).

**Fig 5:**
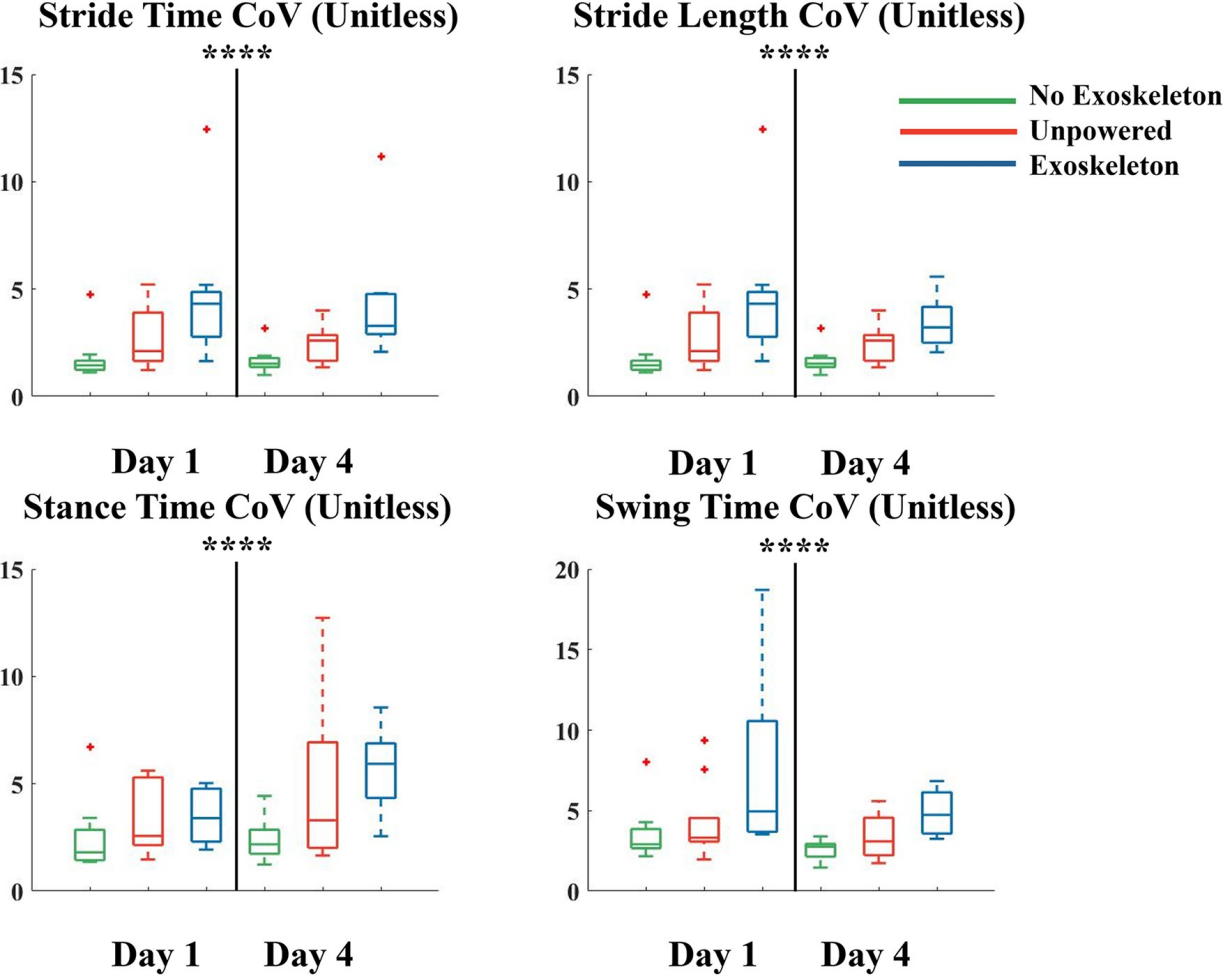
Coefficient of Variation (CoV) presented as a percentage for the stride time, stride length, stance time and stance length while walking on a treadmill on the first (left side of each subplot) and final (right side of each subplot) days of training without the exoskeletons (green), with the exoskeletons on and unpowered (orange), and with (blue) the exoskeleton. There was significantly higher variability in all measures when wearing the exoskeleton compared to not wearing the exoskeleton on both the first and final day of training. Data is represented as a box plot where the central line indicates the median of the data, the top and bottom edges of the box indicate the 25^th^ and 75^th^ percentile, and the whiskers represent the minimum and maximum values. Red crosses indicate outliers in the data. **** indicates significant differences between exoskeleton conditions. There were no significant differences due to the day.

Walking with the exoskeleton inside on the treadmill resulted in higher coefficients of variation compared to not wearing the exoskeletons in stride time (p<0.001), stride length (p<0.001), stance time (p = 0.033), and swing time (p = 0.005) with no significance due to day. In addition, walking with the exoskeletons powered resulted in significantly higher coefficients of variation compared to walking with the exoskeletons unpowered in stride length (p = 0.031), stride time (p = 0.015), and swing time (p = 0.039). There were no significant differences in coefficients of variation between the no exoskeleton and unpowered conditions. Contrary to our hypothesis we did not find significant differences in the measurements between day 1 and day 4 (Fig 5). On day 1 the coefficient of variation of the stride length and stride time was the same because the treadmill was set at a constant speed. The coefficient of variation for both were 5% in the exoskeleton condition and 2% in the no exoskeleton condition, a difference of 91%. On day 4 there was a reduction in the coefficient of variation of stride time and stride length in the exoskeleton condition to 4% and 2% in the no exoskeleton condition, with a difference of 89% between exoskeleton and no exoskeleton condition. On day 1 the coefficient of variation of the stance time was 6% in exoskeleton condition and 3% in the no exoskeleton condition, a difference of 81%. On day 4 there was an increase in the stance time coefficient of variation in the no exoskeleton condition to 6% condition and a decrease to 2% in the exoskeleton condition, with a difference between exoskeleton and no exoskeleton condition of 81%. On day 1 the coefficient of variation of the swing time was 8% in exoskeleton condition and 4% in the no exoskeleton condition, a difference of 72%. On day 4 there was a reduction in the swing time coefficient of variation in the exoskeleton condition to 5% and no exoskeleton condition to 3%, with a difference between exoskeleton and no exoskeleton condition of 62%.

**Fig 6:**
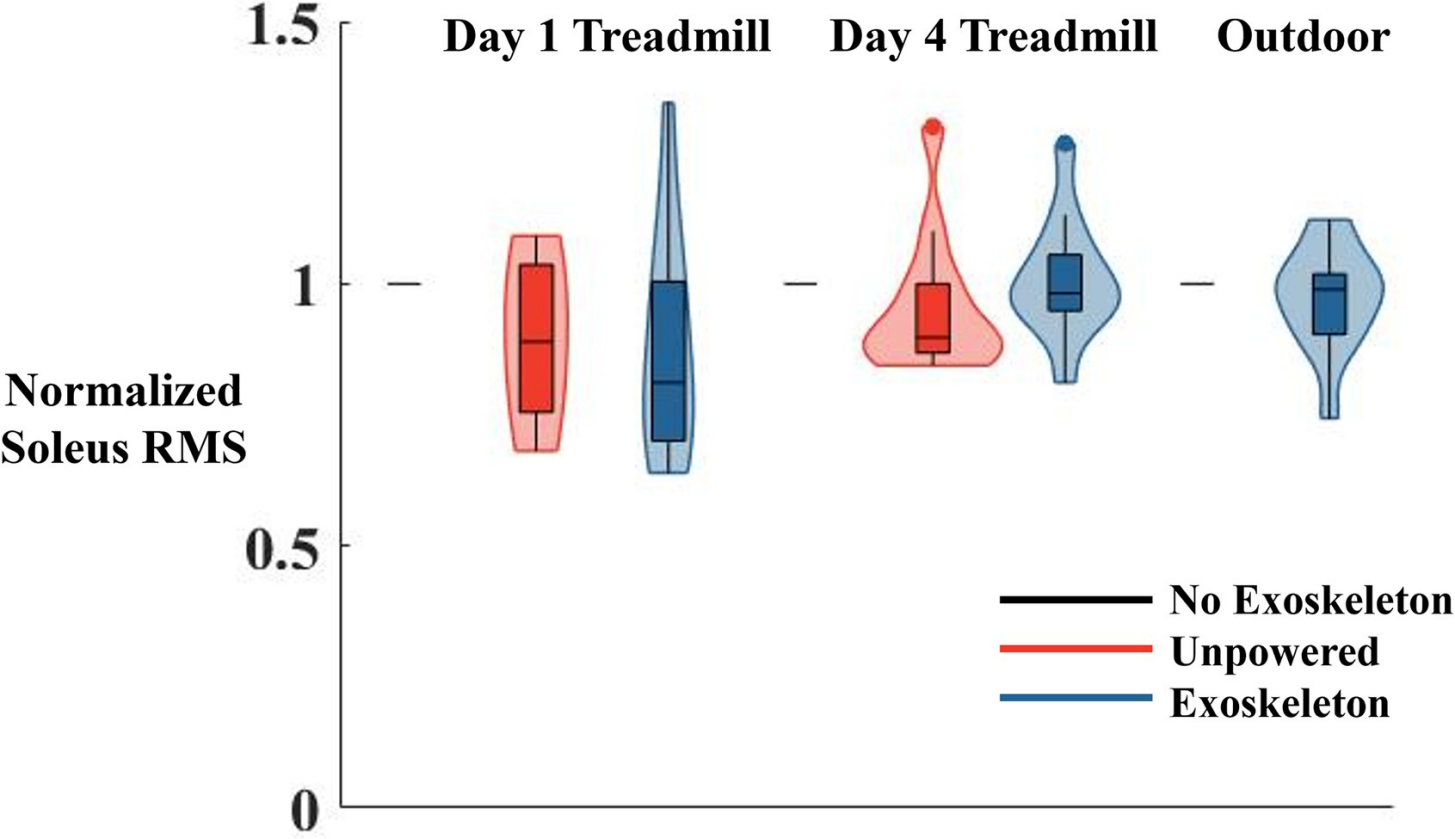
Soleus EMG RMS values for the first day of training (left), the final day of training (middle) and outdoor walking (right) normalized to the no exoskeleton condition in each training condition. There were no significant differences between the no exoskeleton (shown as a line at 1 due to normalization), unpowered exoskeleton (red), and exoskeleton powered (blue) conditions during any of the training paradigms. Figures are represented as violin plots. Within each plot the box indicates the median and first and third quartiles. The shaded area shows the distribution of the data across participants.

There were non-significant differences in the EMG activity across conditions. We present the Soleus as a representative muscle because of its part in the control mechanism (Fig 6). On the first day participants had an average of 12% lower soleus activity in the exoskeleton condition during stance compared to the no exoskeleton condition (p = 0.476), the medial gastrocnemius root mean squared value was 5% less (p = 0.168), and the lateral gastrocnemius root mean squared value was 17% less (p = 0.059). The tibialis anterior RMS was 9% higher after the first day of training (p = 0.185). On the fourth day of training, the soleus activity was 7% higher during the exoskeleton condition than the no exoskeleton condition (p = 0.98), the medial gastrocnemius root mean squared value was 8% more (p = 0.617), and the lateral gastrocnemius root mean squared value was 33% more (p = 0.759). The tibialis anterior RMS was 14% higher after the final day of training (p = 0.617). During the outdoor walking conditions, the soleus activity was 9% lower in the exoskeleton condition than the no exoskeleton condition (p = 0.882), the medial gastrocnemius root mean squared value was 3% more (p = 0.355), and the lateral gastrocnemius root mean squared value was 4% more (p = 0.848). The tibialis anterior RMS was 1% higher after the final day of training (p = 0.952).

## Discussion

As hypothesized, we found significantly higher coefficients of variation of stride length, stance time, and swing time while walking outside both with and without the exoskeletons compared to walking inside on a treadmill. There was an 119% difference in stride length variability when walking with the exoskeletons outside compared to walking with them on the treadmill. There was a 162% difference in stride length variability when walking without the exoskeletons outside compared to on the treadmill. This was the largest increase in variability we found. Contrary to the findings in other studies without exoskeletons, we saw an average of 29% shorter stride lengths walking outside compared to on the treadmill [46,47]. The walking speeds outside (1.2 m/s with the exoskeleton and 1.17 m/s without the exoskeletons) were slightly faster than on the treadmill (1.14 m/s) but the difference was not large or statistically significant. Apart from the slightly larger variability in the stance time during treadmill walking compared to outdoor overground walking, our findings are consistent with other literature without exoskeleton use that shows that treadmill walking leads to less variable gait patterns and walking outside leads to more variable gait [14,31,33]. Studies looking at gait variability and spatiotemporal measures in older adults show that the longer stance times, shorter swing times, and more variable stride times demonstrated on a treadmill may be a way of increasing stability when the participant feels less in control of the environment [47,48]. It may be that walking on a treadmill causes participants to feel less secure compared to walking overground outside. We did not find any correlations between our subjects’ heights and weights with changes in variability in any of our spatiotemporal measures. This may be due to our subjects all being healthy and young. A larger study that has height and weight as controlled variables may show more insight into how they may be related to gait variability.

**Fig 7:**
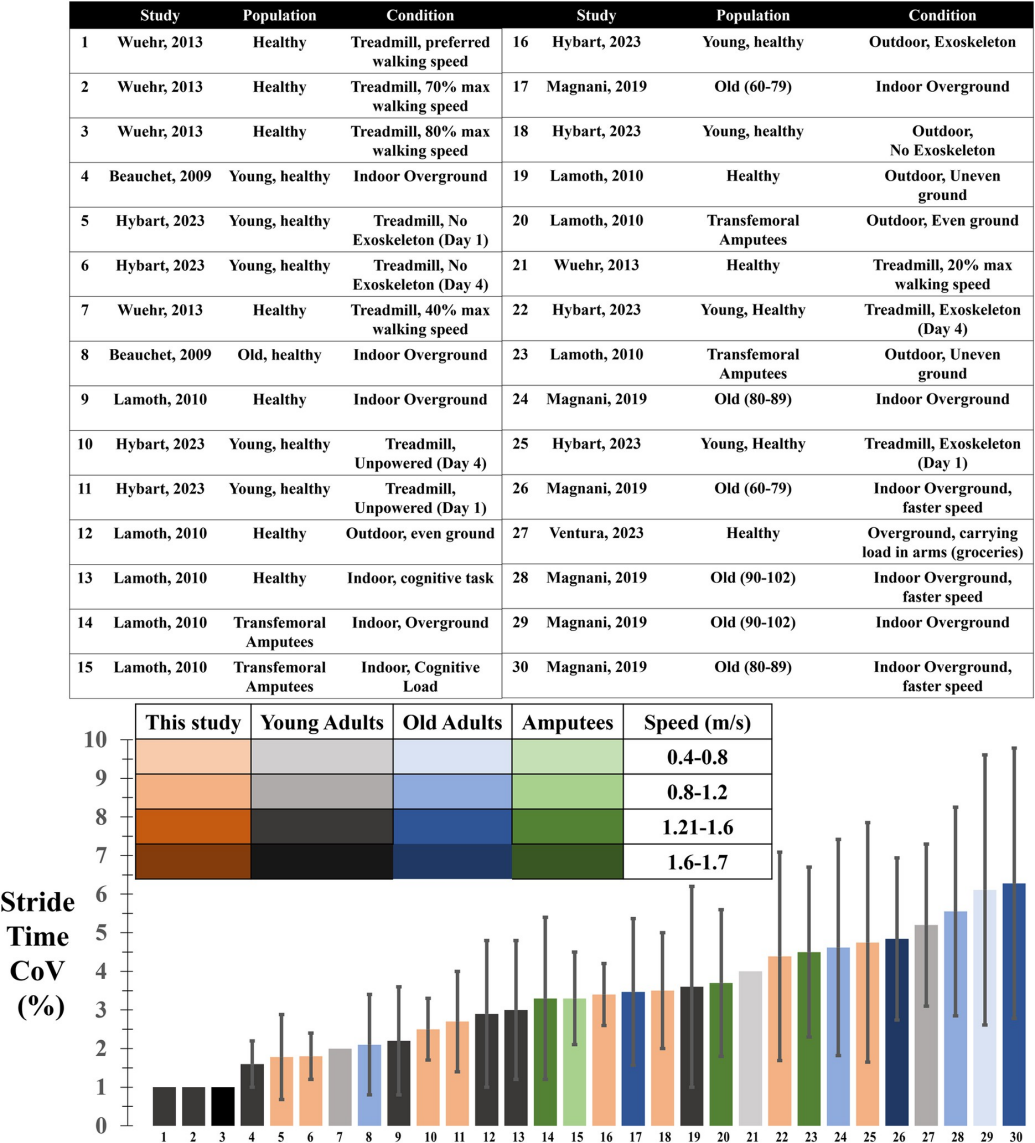
Stride time Coefficients of Variation (CoV) (%) for six studies across different populations. For each bar in the bar graph there is a line in the table that indicates what paper the data is from, what the participant population was, and what the testing conditions were for that bar. In the bar graph the colors indicate the population: Young adults (grey scale), old adults (blue scale), or amputees (green scale). The study presented in this paper is represented in orange. The different shades of each color represent the speed range that the participants walked: 0.4–0.8 m/s (light), 0.8–1.2 m/s (medium-light), 1.2–1.6 m/s (medium-dark), and 1.6–1.7 m/s (dark). Overall, values found for stride time variability in our study were within the range of reported values for a range of populations and conditions. Error bars represent ±1 standard deviation, there was one paper that did not include standard deviation values.

Contrary to our hypothesis, participants had significantly higher coefficients of variation while walking with the exoskeletons compared to without the exoskeletons when walking on the treadmill. Stride time, stride length, swing time, and stance time all had greater variability with the exoskeleton on the treadmill compared to not using the exoskeleton on the treadmill. These data suggest that there was more exploration in walking strategies by the participants when they had the exoskeletons on the treadmill. However, there was no difference in walking with and without the exoskeletons outside. The fact that when walking overground, stride length and swing time variability when using the exoskeletons (14% and 7%, respectively) compared to not using them (17% and 7%, respectively) was similar suggests that the variability due to terrain differences (treadmill vs. outside) has a larger effect than the variability due to exoskeleton assistance. This is further supported by the non-significant changes in muscle activity when walking with and without the exoskeleton overground on the treadmill (Fig 6). There were no differences in the coefficient of variations for any of the spatiotemporal measures due to the day of testing or when walking outside due to the exoskeletons. Overall the stride time variability we found in the present study was within the range of the stride time variability found in previous research that has assessed gait spatiotemporal measures across different experimental data in the literature (treadmill vs overground, load carriage, prosthetic use, and age) (Fig 6) [49–53]. Across these and other studies, the stride time coefficient of variation has been used as the most common measure of gait variability. Fig 7 shows a range of 1% to 7% variability in stride time in various subject populations and walking speeds. In the study presented in this paper we measured a stride time variability of between 2% and 5%. One study assessing young healthy adults walking on a treadmill at a variety of speeds surrounding their preferred walking speed [52] found that at preferred walking speeds and those slightly higher than preferred, participants had the lowest variability in stride time. In contrast, in aging populations, faster walking speeds generally led to larger stride time variability [49]. Besides walking speed, changing the terrain, adding a load, or adding a cognitive task also increased stride time variability. In both a control group and an amputee population, performing a cognitive task while walking overground indoors, led to a slightly slower walking speed and higher variability than walking overground without the cognitive task [51]. This same study found that walking outdoors on even and uneven ground led to higher variability than walking overground inside. In a final study, carrying a load of groceries affected indoor overground gait, and led to a variability twice as large as other studies walking indoor overground without a load [50,53]. Our data represents healthy young adults, walking on a treadmill and overground, without exoskeletons, with exoskeletons worn and unpowered, and with the exoskeletons actively assisting. We found the average stride time variability when walking without the exoskeletons on both the first and final days (2%) to be around the same as the average of the studies where young adults (both control and amputee population) walked on a treadmill and inside overground (2%). When walking outside overground both with and without the exoskeleton, we saw similar stride time variability (4%) compared to when other young adults (both control and amputee populations) walked outside overground (4%). For stride time variability specifically, we found higher values due to walking on the treadmill with the exoskeletons powered than in any other condition, but only significantly different than walking on the treadmill without the exoskeletons. This suggests that the added mass and assistance caused more variable gait when participants walked at a set speed on a treadmill.

Future studies should include tests of indoor overground walking, uneven treadmill walking, as well as incline and decline treadmill walking in addition to the conditions studied in this paper. Having indoor and outdoor conditions on flat, incline, decline and uneven terrains will give a more encompassing view of how people adapt their gait to exoskeletons. A comparison of these exoskeletons on a self-paced treadmill may give more insight into how much of the variability we observed on the treadmill was due to the constrained speed rather than exploration using the exoskeletons. Other papers saw similar stride time variability but higher stride length variability when walking on a self-paced treadmill than we saw in this study [54,55]. Exploring how self-paced treadmills, fixed-pace treadmills, and outdoor overground walking compare when using an exoskeleton will help fit this research better into the current scope of research on gait variability. In addition, studying how variability changes based on the type of exoskeleton controller would give insight into how different control methods may suit different walking scenarios better.

## Conclusions

We tested the hypotheses that individuals using electromechanical ankle exoskeletons under proportional myoelectric control would: 1) have higher variability in gait spatiotemporal measures when walking outside both with and without the exoskeletons compared to walking on a treadmill, and 2) have similar amounts of variability when walking with the exoskeletons compared to without the exoskeletons. In support of our first hypothesis, participants had larger coefficients of variation in stride length, stance time and swing time when walking outside overground compared to on the treadmill. In partial support of our second hypothesis there were no differences in variability when using the exoskeletons outside compared to not using the exoskeletons outside. However, when walking on the treadmill participants had larger coefficients of variation when using the exoskeletons than when not using the exoskeletons. Based on our results, only studying gait variability on a treadmill may lead to the conclusion that exoskeletons elicit significant changes in variability. Walking overground revealed that the exoskeletons do not lead to significant changes in gait spatiotemporal measures or their variability in the real-world. When using exoskeletons intended for use in assistance or rehabilitation the devices should work with the user to adapt to changes in the environment to create a relationship of trust between the human and the machine, rather than adding to the uncertainty of movements. In the context of the literature, we show that variability due to robotic ankle exoskeletons under proportional myoelectric control does not elicit different changes in stride time variability than previously found in other daily living tasks (uneven terrain, load carriage, or cognitive tasks).
